# Feasibility study on the application of a spiking neural network in myoelectric control systems

**DOI:** 10.3389/fnins.2023.1174760

**Published:** 2023-06-12

**Authors:** Antong Sun, Xiang Chen, Mengjuan Xu, Xu Zhang, Xun Chen

**Affiliations:** Department of Electronic Science and Technology, University of Science and Technology of China (USTC), Hefei, Anhui, China

**Keywords:** gesture recognition, electromyography, spike encoding, LIF, SNN

## Abstract

In recent years, the effectiveness of a spiking neural network (SNN) for Electromyography (EMG) pattern recognition has been validated, but there is a lack of comprehensive consideration of the problems of heavy training burden, poor robustness, and high energy consumption in the application of actual myoelectric control systems. In order to explore the feasibility of the application of SNN in actual myoelectric control systems, this paper investigated an EMG pattern recognition scheme based on SNN. To alleviate the differences in EMG distribution caused by electrode shifts and individual differences, the adaptive threshold encoding was applied to gesture sample encoding. To improve the feature extraction ability of SNN, the leaky-integrate-and-fire (LIF) neuron that combines voltage–current effect was adopted as a spike neuron model. To balance recognition accuracy and power consumption, experiments were designed to determine encoding parameter and LIF neuron release threshold. By conducting the gesture recognition experiments considering different training test ratios, electrode shifts, and user independences on the nine-gesture high-density and low-density EMG datasets respectively, the advantages of the proposed SNN-based scheme have been verified. Compared with a Convolutional Neural Network (CNN), Long Short-Term Memory Network (LSTM) and Linear Discriminant Analysis (LDA), SNN can effectively reduce the number of repetitions in the training set, and its power consumption was reduced by 1–2 orders of magnitude. For the high-density and low-density EMG datasets, SNN improved the overall average accuracies by about (0.99 ~ 14.91%) under different training test ratios. For the high-density EMG dataset, the accuracy of SNN was improved by (0.94 ~ 13.76%) under electrode-shift condition and (3.81 ~ 18.95%) in user-independent case. The advantages of SNN in alleviating the user training burden, reducing power consumption, and improving robustness are of great significance for the implementation of user-friendly low-power myoelectric control systems.

## 1. Introduction

Surface electromyography (sEMG) signals, which originate from motor neurons in the spinal cord and can accurately reflect muscle activity, is a common medium for detecting motor intent. Myoelectric pattern recognition is a technique of translating body movements into machine commands via Electromyography (EMG) signals, which is commonly used to implement myoelectric control systems in the fields of prosthetic control and rehabilitation training (Oskoei and Hu, 2007; Xing et al., 2014). Myoelectric pattern recognition usually consists of sEMG signal acquisition and a classifier design. High-density (HD) array electrodes and low-density (LD) separate electrodes are often used to collect sEMG signals. LD sEMG uses a small number of electrodes to record muscle activity with low spatial resolution but low equipment cost. HD sEMG has a higher cost and uses a large number of electrodes to record muscle activity with high spatial resolution, which can capture the distribution of muscle activity and provide more detailed information about muscle activation patterns. With a classifier design, the ideal goal of myoelectric pattern recognition is to implement a general classifier with high generalization capability. However, due to the large individual differences of sEMG signals, the generalization capability of classifiers is often poor in user-independent cases. In fact, most effective myoelectric control systems work in a user-specific mode (Zhang et al., 2020; Chen et al., 2021; Hu et al., 2021; Yu et al., 2021), although training a specific classifier for each user will create a heavy training burden. Even in a user-specific mode, in practical interactive applications, the repeated wearing of the acquisition device will lead to electrode shifts, which will create large differences in the distributions of training data and test data and seriously degrade the performance of the classifier. Therefore, how to design a robust pattern recognition scheme that is insensitive to individual differences and electrode shifts is one of the current research hotspots in the field of myoelectric pattern recognition.

In early research, traditional machine learning algorithms based on manual feature extraction such as support vector machine (SVM) (Cortes and Vapnik, 1995), k-nearest neighbor (KNN) (Cover and Hart, 1967), and linear discriminant analysis (LDA) (Fisher, 1936) have been successfully applied in myoelectric pattern recognition (Du et al., 2010; Phinyomark et al., 2013; Wei et al., 2016). These algorithms were often conducted on LD-sEMG signals in a user-specific mode. The difficulty of applying them to practical myoelectric control systems lies in their low generalization performance to new users. In recent years, the development of artificial neural networks (ANNs) has led to a shift in the research of myoelectric pattern recognition to the field of deep learning (DL) (LeCun et al., 2015). The deep neural network (DNN) based on end-to-end implementation can automatically extract the optimal features with high specificity, making it able to achieve high generalization of myoelectric pattern recognition. Relevant research has verified that DNNs such as convolutional neural networks (CNNs) and long short-term memory networks (LSTMs) can obtain higher recognition accuracy than traditional machine learning methods (Hu et al., 2018; Triwiyanto et al., 2020). In particular, the deep transfer learning method, which combines the feature learning ability of deep learning with the distributed adaptive ability of transfer learning, has been proven to have significant advantages in improving the generalization and reducing the user training burden (Chen et al., 2021; Soroushmojdehi et al., 2022).

Although the research on myoelectric pattern recognition based on DNN has made some progress, considerable progress still needs to be made to meet the actual needs of myoelectric control systems. First, heavy training burden is a prominent problem for the implementation of DNN. Feature learning of complex DNN usually requires large-scale training sets, which requires sufficient training samples to be collected from users, resulting in a heavy user burden. Since pre-training of the source network requires the collection of a large number of samples and the target network requires a certain amount of training data for fine-tuning, even transfer learning cannot fundamentally solve the training burden problem. Second, real-time implementation of DNN is often difficult. DNN often has too many parameters and requires a lot of floating-point multiplication, which leads to high hardware requirements for computational resources and storage space.

In recent years, a third generation of neural network, namely spiking neural network (SNN), has been proposed based on the laws of neuromorphic computing (Izhikevich, 2006). SNN is event-driven and can be combined with event-based sensors to provide an efficient bionic solution for pattern recognition tasks. Specifically, for tactile object recognition based on event-based tactile sensors (Taunyazov et al., 2020), Kang et al. proposed a location spiking neuron based on time-dependent spiking neurons and constructed a hybrid model using both neurons, verifying that the model can better capture the complex spatio-temporal dependencies in event-driven tactile data (Kang et al., 2023). For gesture recognition tasks based on event-based dynamic vision sensors (DVSs) (Brandli et al., 2014), Xing et al. proposed a new spiking convolutional recurrent neural network (SCRNN) architecture, which used convolutional operations and recursive connectivity to maintain spatial and temporal relationships in event-based sequential data and achieved 96.59% accuracy in 10-class gesture recognition and 90.28% accuracy in 11-class gesture recognition (Xing et al., 2020). From the perspective of myoelectric pattern recognition, temporal EMG signals also can be mapped to spike events using specific encoding for pattern recognition using event-driven SNN. In particular, the characteristics of SNN make it possible to achieve a high generalization, low training burden, and low power consumption myoelectric control system. First, SNN uses the biological mechanism of spike neurons to process sequential spikes, making it naturally advantageous when processing physiological signals such as electroencephalogram (Al Zoubi et al., 2018) and functional magnetic resonance imaging (Sengupta et al., 2018) with low training burden; second, SNN performs pattern recognition based on spike events space, which makes it insensitive to amplitude variations due to individual differences and electrode shifts; third, SNN can be implemented with adding operations only due to its binary mechanism, which can greatly reduce computational power and storage resources (Donati et al., 2019; Cheng et al., 2021).

Some scholars have actively applied SNN to myoelectric pattern recognition. For instance, Cheng et al. designed a Leaky-integrate-and-fire (LIF) neuron-based fast spike discharge time search algorithm, constructed a pre-trained sub-network SNN, and obtained 97.4% classification accuracy for eight gestures (Cheng et al., 2021). Garg et al. used a LIF neuron to construct a spiking reservoir with a biologically inspired topology, and obtained the classification accuracies of 89.72 and 70.6% for the 8-chanel EMG datasets with three gestures and five gestures, respectively (Garg et al., 2021). Ma et al. (2020) implemented a spiking recurrent neural network (SRNN) on the Dynamic Neuromorphic Asynchronous Processor (DYNAP) (Moradi et al., 2018), using Spike-Timing Dependent Plasticity (STDP) and soft Winner-Take-All (WTA) for network training, and obtained over 85 and 55% classification accuracy on an 8-chanel EMG dataset with three gestures and the Ninapro dataset (Atzori et al., 2014) with five gestures, respectively (Ma et al., 2020). Tian et al. used the adaptive weight mapping method to convert CNN to spiking-CNN. For 10-channel three-gesture recognition tasks, the gesture recognition accuracy is 85.7% (Tian et al., 2023). Donati et al. implemented a single hidden layer feed-forward SNN with adaptive exponential LIF neurons on a multicore neuromorphic chip and obtained 74% recognition accuracy for a three-gesture recognition task (Donati et al., 2019). However, although existing studies have validated the effectiveness of SNN for myoelectric pattern recognition, they lack comprehensive consideration of user training burden, power consumption, and generalization capability to individual differences and electrode shifts. In other words, the application of SNN in actual myoelectric control systems needs to be deeply explored.

In the above related studies, SNN-based myoelectric pattern recognition has usually focused on spike encoding method, neuron models, network topology, and learning algorithms. In this paper, in order to explore the feasibility of applying SNN to actual myoelectric control systems, we investigate a SNN-based myoelectric pattern recognition scheme. Unlike other studies, the innovation or main contribution of this study is the attempt to explore the problems of training burden, robustness, and power consumption of pattern recognition for actual myoelectric control systems. To this end, the adaptive temporal contrast encoding method is adopted to alleviate EMG distribution differences caused by electrode shifts and individual differences; the LIF neuron is improved by combining voltage and current decay effects to improve the feature extraction ability, and the adaptive threshold encoding parameters and LIF neuron release thresholds are determined experimentally to balance recognition accuracy and power consumption as much as possible. By comparing this with CNN, LSTM, and LDA, the validity of the proposed SNN in reducing training burden, alleviating the influence of electrode shifts and individual differences, and lowering power consumption has been verified.

## 2. Materials and methodology

**Figure 1:**
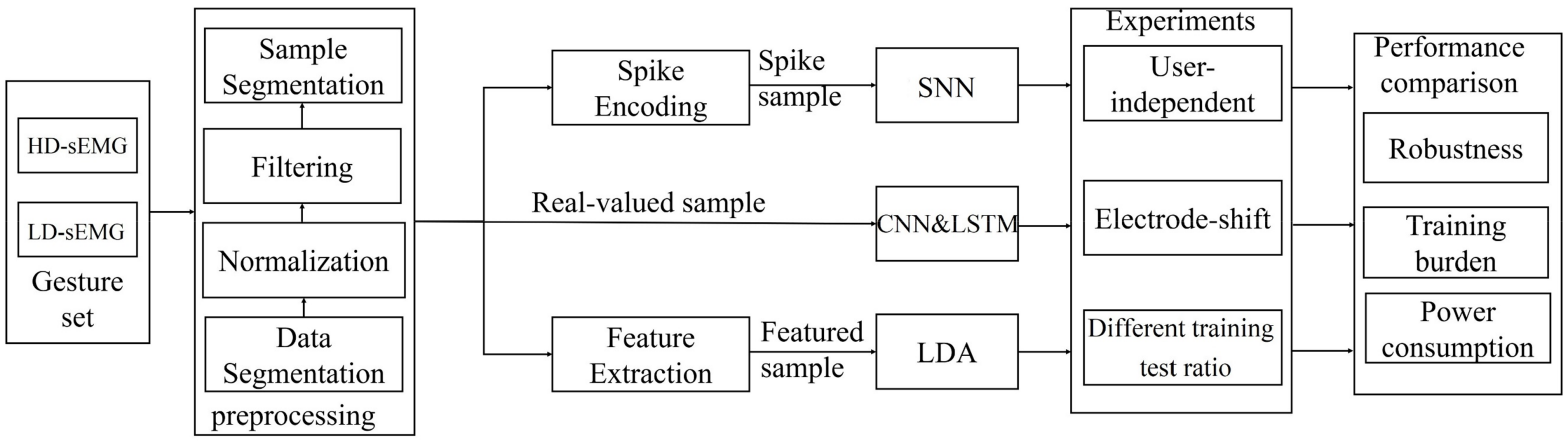
Block diagram of the research route.

Figure 1 presents the research route of the proposed SNN-based myoelectric pattern recognition. It mainly includes EMG sample generation, design of SNN, CNN, LSTM, and LDA classifiers, gesture recognition experiments under user-independent case, electrode-shift case and different training test ratios, and performance analysis. Each part is described in detail below.

### 2.1. Gesture databases

**Figure 2:**
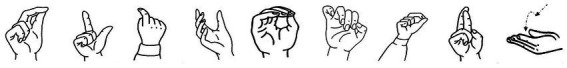
9 kinds of gestures.

**Figure 3:**
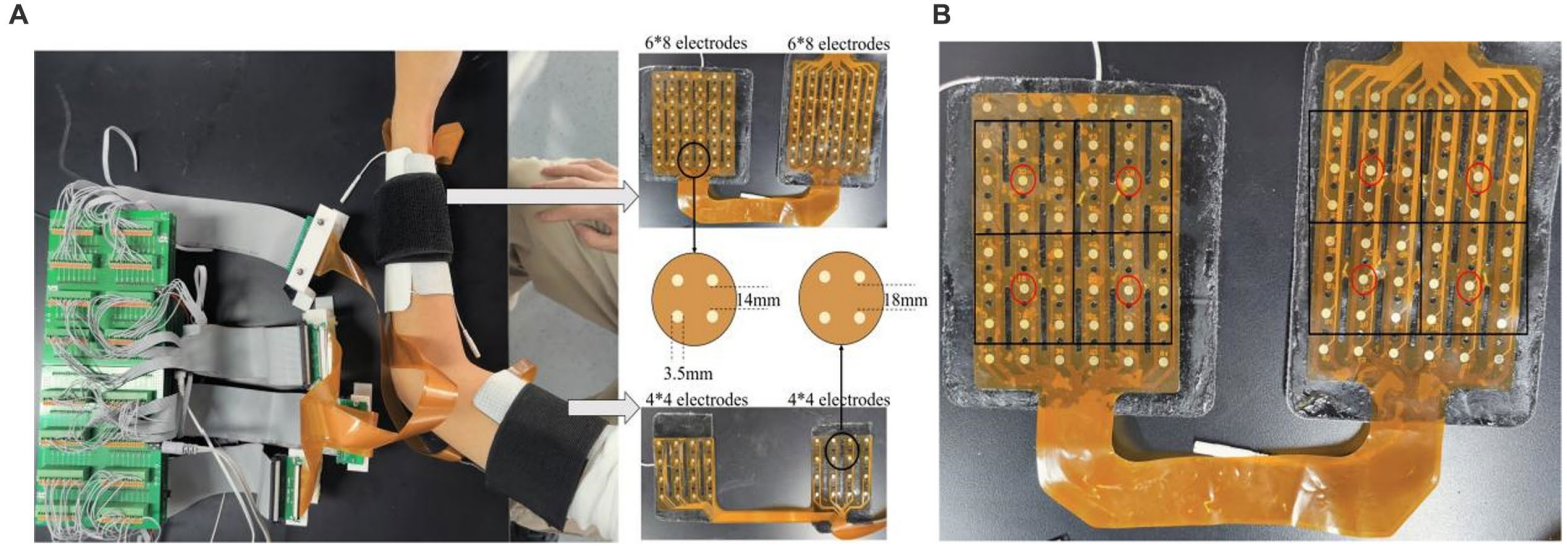
**(A)** The acquisition device and **(B)** the selection method of 8-channel electrodes.

This study takes the 128-channel HD-sEMG database established in our previous studies (Hu et al., 2021) as the target dataset. It consists of HD-sEMG data with nine gestures (Figure 2) and five electrode-shift positions. Eight participants (five men and three women, aged 24–35) participated in the data collection. The acquisition device (Figure 3A) consists of two 48-channel (8 × 6) and two 16-channel (4 × 4) electrode arrays, both with an electrode diameter of 3.5 mm. The electrode spacing is 14 mm and 18 mm for the 48-channel and 16-channel arrays, respectively. The two 48-channel electrode arrays are used to acquire signals from the forearm extensor and flexor muscles, and the two 16-channel arrays are used to acquire signals from the biceps and triceps muscles, respectively. The signal acquisition method is unipolar, i.e., the signal of each channel is the potential difference between the acquisition electrode where the channel is placed and the reference electrode located on the back of the right hand. The sampling frequency is 1 KHz.

All participants were informed of the experimental procedures and signed their informed consent approved by the Ethics Review Committee of First Affiliated Hospital of Anhui Medical University (No. PJ 2014-08-04). During the data collection experiment, participants sat comfortably on a chair with their right arm on a table. The skin on the front and back of the forearm was wiped with alcohol and coated with conductive paste. The rules for performing gestures were as follows: the first 2 s were the beginning phase, which involved relaxing the arm on the table and lifting the elbow to execute the gesture; the middle 2 s were the steady-state phase where the hand shape and strength remain unchanged; the last second was the end phase, when the muscles relaxed and the elbow returned to the table at free speed. Before data collection, participants were asked to practice completing one gesture action within 5 s until they become proficient.

The data were collected in five different trials over 2 ~ 3 days. In each trial, participants performed all gestures at a comfortable level of moderate effort, and repeated each gesture eight times. Since the five trials for the data acquisition were performed at different times, re-wearing of the acquisition device inevitably created electrode shifts. It is worth mentioning that the electrodes could be moved appropriately in the medial/lateral or distal/proximal directions during the wearing of the device, but they could not be rotated. To do this, participants were asked to keep their palm facing upwards with all five fingers together while straightening the fingers and arm, and the left and right edges of the electrode piece were always parallel to the middle finger, ensuring that the electrode piece did not rotate.

To verify the performance of the proposed SNN on both HD-sEMG and LD-sEMG signals, LD-sEMG dataset with 8-channels was also established by the following steps: Because the forearm extension and flexor muscles usually have richer activation modes during gesture execution, we selected eight channels from the two 48-channel electrode arrays covering the two muscles. As shown in Figure 3B, the selection rule was to choose a 6×6 electrode matrix from a 6×8 electrode array, then divide it into four 3×3 sub-matrices, and finally select the center electrode of each sub-matrix.

### 2.2. Gesture sample generation

Since different pattern recognition schemes involved in this study have different requirements for input samples, gesture samples are generated through the following steps:

Step 1: raw EMG signal is pre-processed through amplitude-based data segmentation, filtering, and normalization to obtain a 5 s EMG active stream corresponding to each repetition. At first, a few channels (about 2 ~ 3 channels), whose signal amplitudes are beyond the reasonable range, are discarded and replaced by the average value of adjacent channels; then, the signals are segmented based on amplitude threshold. When the signal amplitude rises or falls to about 10% of the peak, the corresponding time point is considered to be the beginning or end of an active data segment, respectively. All active data segments are resampled into 5,000 points (corresponding to 5 s); finally, the signal of the active data segment is filtered using a 20–500 Hz, 50th-order finite spike response (FIR) bandpass filter, and normalized to 0 ~ 1 with respect to the Min-Max of each segment.
Step 2: sliding window sample segmentation is applied to the active data segment of each EMG stream to obtain real-valued samples that can be directly input into CNN and LSTM. For an EMG stream corresponding to one gesture repetition, a sliding window (length: 100 ms, increment: 50 ms) is adopted to segment the data during the stabilization phase (2nd to 4th seconds, 2000 ms) to obtain 39 real-valued EMG samples. According to the operation, each participant can obtain 5(trials)×9(gestures)×8(repetitions)×39(windows)=14,040 samples. The size of a real-valued sample is 128 or 8 (channels) × 100 (time steps) for the HD-sEMG dataset or the LD-sEMG dataset, respectively.
Step 3: for real-valued samples, four time-domain features, namely mean amplitude value (MAV), variance (VAR), waveform length (WL), and zero-crossing (ZC), are calculated for each channel to get the featured samples. Therefore, the size of a featured sample is 128 or 8 (channels) × 4 (Number of features).
Step 4: the real-valued samples are encoded to spike samples suitable to SNN as described in the following section.

### 2.3. Temporal encoding based on adaptive threshold

The commonly used spike encoding methods are rate encoding (deCharms and Merzenich, 1996) and temporal encoding (Bohte, 2004). Compared with rate encoding, temporal encoding focuses more on the differences in temporal structure, and the temporal logic between spikes is considered to have the potential to encode important information. To take full advantage of the ability of SNN to process temporal signals, temporal encoding is applied in this study.

**Figure 4:**
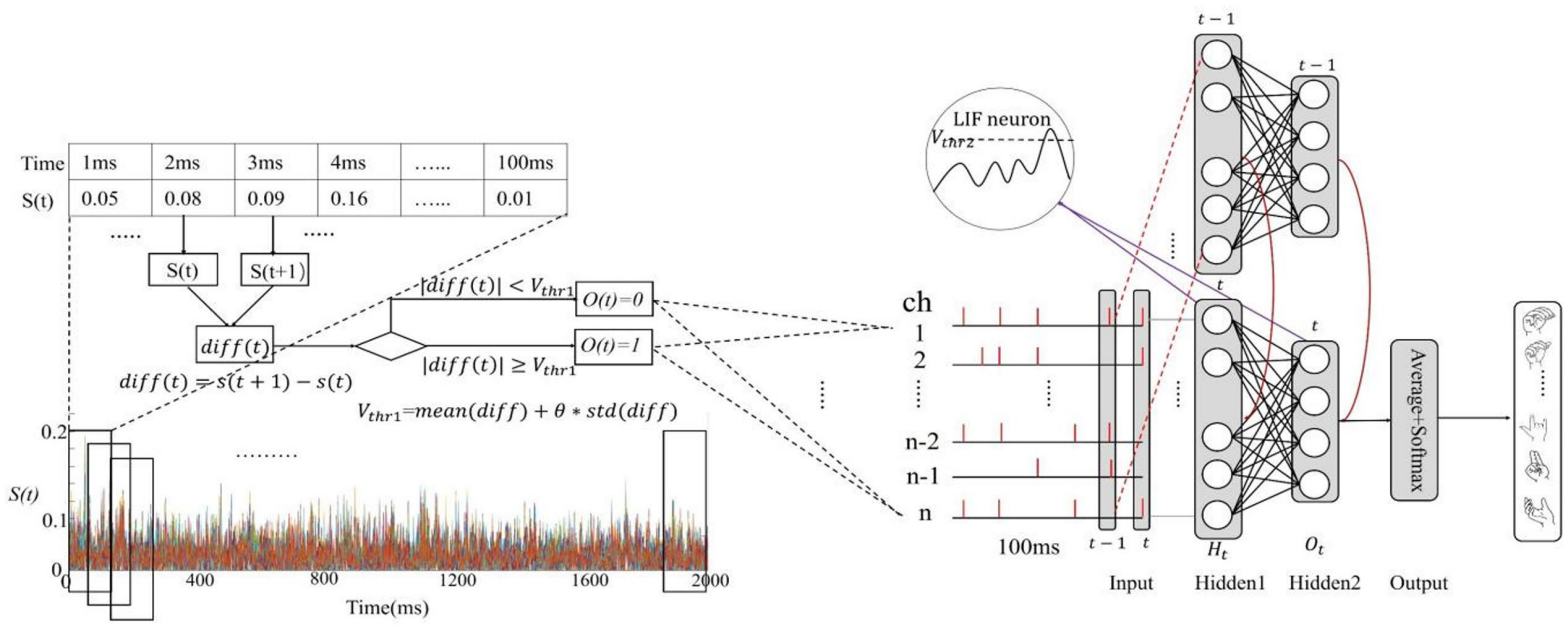
Spike encoding and the architecture of the proposed SNN.

Temporal encoding can be implemented by the temporal contrast algorithm (Petro et al., 2019), which can track the temporal changes of the signal amplitude using the spike. In this study, a temporal contrast algorithm, namely incremental encoding, is adopted. As shown in Eqs. (1), (2), and Figure 4, for a EMG real-valued sample s(t) , first record the signal change at adjacent time points as diff(t) , then compare the absolute value of diff(t) with a threshold $V_{thr1}$ to determine the spike issuing and obtain spike samples o(t) . The encoding time window (T) is equal to the time length of EMG sample.


(1) diff(t)=s(t+1)−s(t)

(2) $$o\left(t\right)=\{\begin{array}{c} 0,\left|diff\left(t\right)\right|<V_{thr1}\;\\ 1,\left|diff\left(t\right)\right|\geq V_{thr1} \end{array}$$

Obviously, the sparsity of the spike samples depends on $V_{thr1}$ . A common threshold selection method is to determine a fixed threshold from experience (Donati et al., 2019; Garg et al., 2021). This method tends to encode signal differences caused by external adverse factors as spikes, which has weak immunity to interference. Relatively, the adaptive threshold selection method presented in references (Petro et al., 2019; Ma et al., 2020) has better anti-interference ability. As shown in Eq. (3), the threshold is determined by the mean and standard deviation of the signal differences ( diff(t)) , where θ is the parameter that regulates the size of the threshold. The adaptive threshold selection method gives a threshold for each sample by its own distribution, which can effectively overcome the wrong spikes caused by external factors and be suitable for electrode shifts and individual differences.


(3) $$V_{thr1}=mean\left(diff\left(t\right)\right)+\theta \times std\left(diff\left(t\right)\right)\;$$

### 2.4. SNN with LIF neuron

#### 2.4.1. Improved LIF neuron based on voltage and current decay effects

LIF neurons simulate the dynamic processes of neurons with resistor-capacitance circuit formulas (Delorme et al., 1999). During operation, the input current I charges the capacitor. When the capacitor voltage exceeds a threshold, the capacitor generates a discharge phenomenon through leakage current. The differential formula for membrane voltage dynamics is expressed by Eq. (4), where U(t) represents membrane voltage of the neuron, $\tau_{mem}$ represents membrane time constant, I(t) is input synaptic current integrated with input spike, and R is membrane resistance.


(4) $$\;\tau_{mem}\frac{dU\left(t\right)}{dt}=-U\left(t\right)+RI\left(t\right)$$

For the LIF neuron in layer l with index i , the membrane voltage can be described in more explicit difference Eqs. (5) and (6), where τ=e−1τmem , l and l−1 represent the current layer and the last layer respectively, j represents the jth neuron of last layer, R is set to unit resistance, $S_{j}^{l-1}\left(t\right)$ is the input spike, and $w_{ij}$ is the synaptic weight from the jth neuron in the last layer ( l−1 ) to the ith neuron in the current layer ( l ).


(5) $$U_{i}^{\left(l\right)}\left(t\right)=\tau U_{i}^{\left(l\right)}\left(t-1\right)+RI_{i}^{\left(l\right)}\left(t\right)$$

(6) $$\;I_{i}^{\left(l\right)}\left(t\right)=\underset{j}{\sum }w_{ij}S_{j}^{l-1}\left(t\right)$$

It can be seen that the traditional LIF neuron determined by Eqs. (5) and (6) only considers the dynamic attenuation of membrane voltages. In fact, the biological synaptic current I itself follows specific time dynamics. To improve the biological rationality of neurons, further modeling for synaptic currents is considered. Referring to reference (Neftci et al., 2019), a simplified first-order approximation depicted in Eq. (7) can be adopted to model exponentially decaying current, where S(t) is the input spike, W is the input synaptic weight matrix, and $\tau_{syn}$ is the synaptic decay time constant.


(7) $$\;\frac{dI\left(t\right)\;}{dt}=-\frac{I\left(t\right)}{\tau_{syn}}+WS\left(t\right)$$

Thus, the Eq. (6) can be rewritten as Eq. (8), where μ = e−1τsyn . The improved LIF neuron is determined by Eqs. (5) and (8). It can be seen that the state of the neuron is given by the membrane voltage and synaptic current cyclically decaying in time step together. Note that (0< μ and τ <1) represent degree of leakage of voltage and current, respectively. $\tau_{syn}$ and $\tau_{mem}$ are equal to 5 and 10.


(8) $$\;I_{i}^{\left(l\right)}\left(t\right)=\mu I_{i}^{\left(l\right)}\left(t-1\right)+\underset{j}{\sum }w_{ij}S_{j}^{l-1}\left(t\right)$$

When $U_{i}^{\left(l\right)}\left(t\right)$ reaches the firing threshold $V_{thr2}$ , the neuron emits a spike according to Eqs. (9) and (10). Then the neuron enters the refractory period and the membrane voltage is reset by subtracting the reset voltage as shown in Eq. (11), where the reset voltage is equal to $U_{i}^{\left(l\right)}\left(t\right)$ multiplied by a penalty parameter p . It should be pointed out that the membrane voltage is not reset to 0 but a negative value, which can effectively suppress the continuous disbursement. In this study, p is taken as 1.5.


(9) $$\;S_{i}^{\left(l\right)}\left(t\right)=h\left(\;U_{i}^{\left(l\right)}\left(t\right)-V_{thr2}\right)$$

(10) $$\;h\left(x\right)=\{\begin{array}{c} 0,x<0\;\\ 1,x\geq 0 \end{array}$$

(11) $$U_{i}^{\left(l\right)}\left(t\right)=U_{i}^{\left(l\right)}\left(t\right)\left(1-p\right)$$

#### 2.4.2. Structure of the SNN

The basic framework of SNN as shown in Figure 4 has been constructed. The SNN consists of the input layer, two SNN hidden layers, and an output layer. The input layer consists of 128 or eight neurons receiving 128 or eight channels of EMG spike every time step. The two hidden layers are to extract the spatiotemporal features by LIF neurons. The spike neurons typically require the calculation of multiple time steps, referred to as the integration time window, which is commonly equal to the encoding time window (T = 100). The output layer calculates the average membrane voltage of the second hidden layer over the time dimension and uses softmax to obtain the gesture classification as shown in Eq. (12). The number of neurons in the first hidden layer is determined experimentally and the number of neurons in the second hidden layer is equal to the number of classification gestures. SNN uses cross entropy to obtain the loss function, and the training algorithm of SNN is the back propagation algorithm of alternative gradient (Neftci et al., 2019).


(12) $$o=\mathrm{softmax}\left(\frac{1}{T}\sum U\left(t\right)\right)$$

### 2.5. The definition of spike release rate

The spikes transmitted in SNN consist of the spikes of gesture samples and the output spikes of the neuron. To better evaluate the power consumption of SNN, the spike release rate ( SRR ) is uniformly defined as Eq. (13). For the gesture spike sample, *N* is the number of channels (128 or 8), *T* is the encoding time window, and *n* is the number of spikes calculated by counting the instances of 1 in the sample. For neurons in a certain layer, SRR represents the average of all neurons firing spikes in an integration time window. Specifically, *T* is the integration time window, *N* is the number of spike neurons, and *n* is the number of spikes issued by *N* neurons within *T*. Theoretically, the smaller SRR , the lower the power consumption. However, smaller SRR may lead to the degradation of accuracy. Therefore, the relationship between SRR and gesture recognition accuracy should be explored to strike a balance.


(13) $$SRR=\frac{n}{T\times N}$$

### 2.6. The contrast classifiers and performance evaluation index

#### 2.6.1. LSTM, CNN, and LDA

This study also uses LSTM, CNN, and LDA for comparison. The network structure of LSTM is determined based on SNN, namely consisting of an input layer, an LSTM layer, and a fully-connected (FC) layer. As for CNN, Chen et al. has designed a ConvNet for myoelectric pattern recognition (Chen et al., 2021). Since the form of EMG signals in this study is consistent with their work, the same structure is adopted. The input of the ConvNet is sEMG image with the size of C×H×W , where C=100 and H and W is the row and column width of the input. The 128-channel EMG signals are reshaped into 16 × 8. The 8-channel EMG signals are reshaped into 4 × 2. The CNN contains two convolutional blocks and a FC layer with softmax . In the two convolutional blocks, the convolutional layers consist of $C_{1}($ 32) and $C_{2}($ 16) filters respectively, with padding to the same output dimensions. The size of the filters is $k_{1}$ * $k_{2}$ with a span of 2, where $k_{1}$ =2 and $k_{2}$ =2. The batch normalization (BN) layer is used to accelerate the convergence of the network and prevent the gradient from disappearing (Ioffe and Szegedy, 2015). The maximum pooling layer is used to further extract effective features and reduce the dimensionality of the features. The output of the last convolutional block is spanned into a one-dimensional vector by the spreading layer. The number of neurons of the FC layer is the number of gestures. LDA is a classical supervised data dimensionality reduction method proposed by Fisher (1936). As a common machine learning algorithm, it is the most widely used classifier in the field of myoelectric pattern recognition. In this paper, the EMG featured samples with four time-domain features are directly input into LDA for gesture recognition.

#### 2.6.2. Performance evaluation index

In this study, we use the inference power, i.e., the power consumed to perform a gesture classification, to measure the power consumption. The inference power is calculated in terms of the number of accumulation (AC) and multiply-accumulate (MAC) operations. In ANN, neurons rely on floating-point matrix multiplication, which require too many MACs. In SNN, due to binary characteristics of the spike, spike neurons only require ACs for matrix multiplication, and only a few MACs are used to update membrane voltage. The literature (Horowitz, 2014) reported that 32-bit floating-point MAC consumes 31 times more power than AC on 45 nm CMOS25. Combining the structures of SNN, LSTM, and CNN, the calculation formulas of the inference power are shown in Table 1, where 0.1 and 3.1 represent relative power of AC and MAC, and T is encoding or integration time window. For encoding, power consumption is mainly contributed to by the calculation of mean and standard deviation, and ch is the number of channels of EMG signal. For SNN, $SRR_{1}$ and $SRR_{2}$ are the SRR of the input sample and the output of the hidden layer respectively, and i , h , and o are the number of input neurons and the two SNN hidden layers neurons, respectively. For LSTM, the inference power is contributed to by the LSTM layer and the FC layer. For CNN, the inference power is contributed by the two convolutional layers and the FC layer.

**Table 1:** The calculation formulas for inference power.

Encoding	Mean	Std
	( T∗0.1+3.1)∗ch	(T∗0.1+(T+1)∗3.1)∗ch
SNN	**SNN Layer1**	**SNN Layer2**
	$T*\left(\begin{array}{c} SRR_{1}*ih*0.1+\\ 2*h*3.1 \end{array}\right)$	$T*\left(\begin{array}{c} SRR_{2}*oh*0.1+\\ 2*o*3.1 \end{array}\right)$
LSTM	**LSTM layer**	**FC Layer**
	T∗(4∗(ih+hh)∗3.1)	T∗ho∗3.1
CNN	**2 CNN layers**	**FC layer**
	$\left(\begin{array}{c} k_{1}*k_{2}*H*W*\\ \left(C*C_{1}+C_{1}*C_{2}\right) \end{array}\right)*3.1$	$3.1*H*W*C_{2}*o$

Recognition accuracy is defined as the ratio of the number of EMG samples correctly recognized to the number of all input samples. The statistical analysis is carried out on IBM SPSS Statistics (Version 26), and the significance level is 5%. Inference delay time typically refers to the time it takes for a model to process and predict input data. It is measured as the time from when the model receives the input data to the output of the prediction result. The training burden is evaluated in terms of user burden, which refers to the amount of data that the user needs to collect. In our paper, the number of gesture repetitions that need to be included in the training set are used as a measure of user training burden.

## 3. Results and analyses

In this study, five types of gesture recognition experiments are carried out. The first experiment is used to determine the network structure and hyper-parameters. The second experiment is designed to determine the encoding parameter and neuron release threshold. The third and fourth experiments are carried out to verify the feasibility of the SNN-based myoelectric pattern recognition in reducing user training burden, mitigating the adverse effects of electrode shifts and individual differences. The last experiment is conducted to demonstrate the superiority of the adaptive threshold encoding and the LIF neuron improved by the voltage–current decay effects.

### 3.1. The determination of network structure and hyper-parameters

The determination of network structure mainly refers to the number of neurons in the hidden layer of SNN and LSTM. The hyper-parameters contain optimizers, batch sizes, and learning rates of three networks. The batch size is set to 1/8 of the training samples. For SNN, the stochastic gradient descent (SGD) (Amari, 1993) is chosen as the network optimizer. For LSTM and CNN, the adaptive moment estimation (Adam) (Kingma and Ba, 2014) is chosen as the network optimizer. The optimal learning rates of SNN, LSTM, and CNN are chosen as 0.1, 0.01, and 0.01, respectively.

The determination experiments are carried out on the HD-sEMG and LD-sEMG dataset, respectively. The encoding parameter θ and neuron threshold $V_{thr2}\;$ of SNN are set to 0and2 . Since one of the major goals is to highlight the advantages of SNN with lower training burden, a small-sample training approach is adopted. Specifically, for the generated spike samples, the training sets and testing sets are divided as follows: one repetition of each gesture is randomly selected to form the training set and the other seven repetitions are used to form the testing set. The experiments are carried out under SNN with different numbers of hidden layer neurons (50, 100, and 150 for 128 channels; 5, 20, and 50 for eight channels). All participants are enrolled in the experiments. As shown in Table 2, the average recognition accuracies of the testing set increase slightly with the number of neurons. Since the increase in the number of neurons leads to the dramatic increase in network training parameters and power consumption, the hidden layer neurons of SNN are set to 100 for 128-channel samples and 20 for 8-channel samples by weighing recognition accuracy and network parameters. As for LSTM, in order to make the structure identical and comparable, the number of hidden layer neurons is consistent with SNN.

**Table 2:** The recognition accuracies (%) under different number of neurons.

Number of hidden neurons	Trainable parameters	Database	
		HD-sEMG	LD-sEMG
50	6,909	91.21 ± 2.23	/
100	13,809	93.50 ± 2.56	/
200	27,609	94.12 ± 2.01	/
10	1,389	/	69.56 ±5.07
20	2,769	/	75.71 ±6.71
50	6,909	/	76.47 ± 5.28

### 3.2. The determination of adaptive threshold encoding parameter θ and LIF neuron release threshold $V_{thr2}$

**Figure 5:**
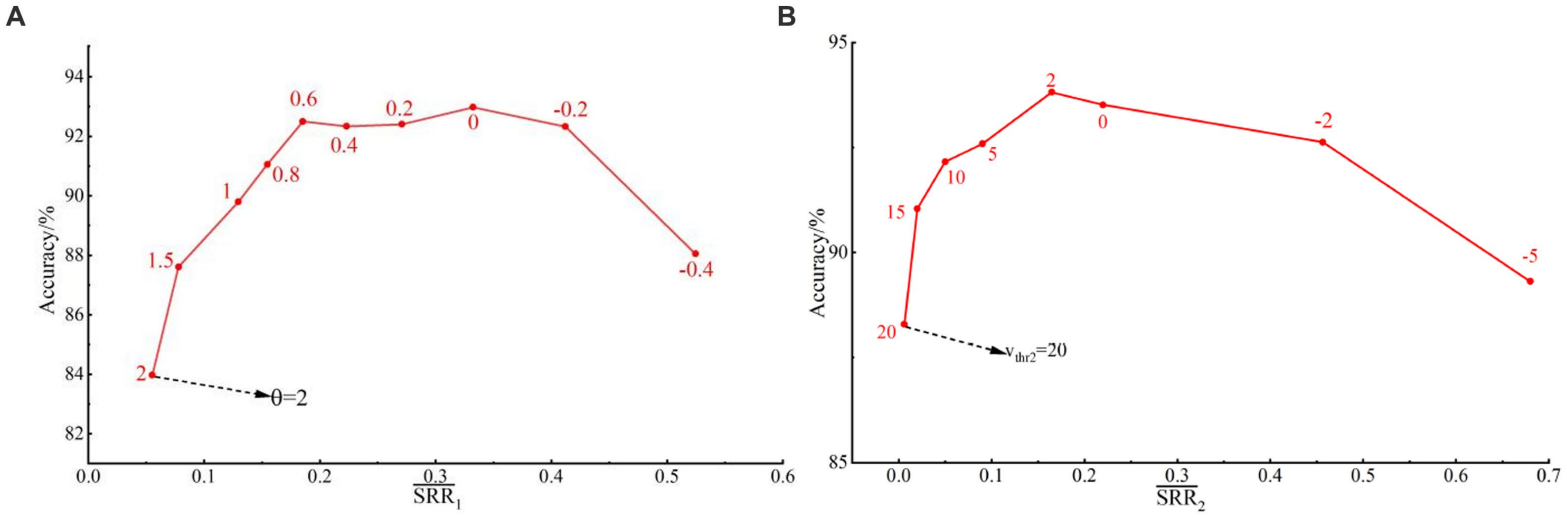
**(A)** The relationships between $\bar{SRR_{1}}$ , θ, and average recognition accuracies; **(B)** The relationships between $\bar{SRR_{2}},V_{thr2}$ , and average recognition accuracies.

The encoding parameter θ and neuron release threshold $V_{thr2}$ are determined experimentally by balancing recognition accuracy and SRR . Since the encoding parameters and firing behavior of single neurons are not affected by the number of channels and neurons, the two parameters are determined only by HD-sEMG dataset. Under determination experiments of θ , the $V_{thr2}$ for both neurons of hidden layers is set to 2, and the θ is traversed in the order of (−0.4 → -0.2 → 0 → 0.2 → 0.4 → 0.6 → 0.8 → 1 → 1.5 → 2). For the generated spike samples, the training set and testing set are also divided into 1:7. $\;\bar{SRR_{1}}$ is the average SRR on all the testing spike samples. When *θ* changes in the above range, the range of $\bar{SRR_{1}}$ is (0.05 ~ 0.55). Figure 5A shows the relationships between $\bar{SRR_{1}}$ , *θ*, and average recognition accuracies. It can be observed that the recognition accuracy increases first and then decreases with the increase of $\bar{SRR_{1}}$ . When $\bar{SRR_{1}}$ falls within the range of (0.2 ~ 0.4), we obtain relatively high and stable gesture recognition accuracies. Corresponding to the range of $\bar{SRR_{1}}$ , the range of θ is (−0.2 ~ 0.6). Therefore, this study takes θ as 0.6 to get a lower SRR in the following experiments.

The determination experiments of $V_{thr2}$ are carried out by the same training test ratios. The $V_{thr2}$ for two SNN layers is set as (−5 → -2 → 0 → 2 → 5 → 10 → 15 → 20). Since only the issued spikes of LIF neurons in the first hidden layer are involved in the information transfer, only SRR of the first hidden layer needs to be considered. $\bar{SRR_{2}}$ is the average SRR input for all test set samples. When $V_{thr2}$ changes in the above range, the range of $\bar{SRR_{2}}$ is (0.006 ~ 0.7). Figure 5B gives the relationships between $\bar{SRR_{2}}$ , $V_{thr2}$ , and average recognition accuracies. When $\bar{SRR_{2}}$ falls within the range of (0.05 ~ 0.4), we obtain satisfactory gesture recognition accuracies. The corresponding range of $V_{thr2}$ is (0 ~ 10). Therefore, this study takes $V_{thr2}\;$ =10 to get a lower spike release rate.

### 3.3. The inference power consumption and delay time of different networks

In this section, the inference power can be calculated on HD-sEMG dataset by formulas shown in Table 1, where $SRR_{1}$ and $SRR_{2}$ are replaced by $\bar{SRR_{1}}$ and $\bar{SRR_{2}}$ and determined by θ=0.6 and $V_{thr2}$ =10, respectively. Table 3 shows the inference power and latency for different networks. The power and delay of encoding and network are considered together for SNN. Compared to LSTM and CNN, the SNN has the absolute advantages of low power consumption and latency. The inference latency of SNN, LSTM, and CNN are 0.073 s, 0.126 s, and 0.133 s, respectively. The inference power of LSTM and CNN are 211.51 and 43.61 times higher than SNN, respectively.

**Table 3:** The inference power and latency(s) of the three networks.

	Encoding + SNN	LSTM	CNN
Inference power	13.64*10^4^	2855.1*10^4^	594.9*10^4^
Inference latency	0.073	0.126	0.1334

### 3.4. Gesture recognition results under different training test ratios

**Figure 6:**
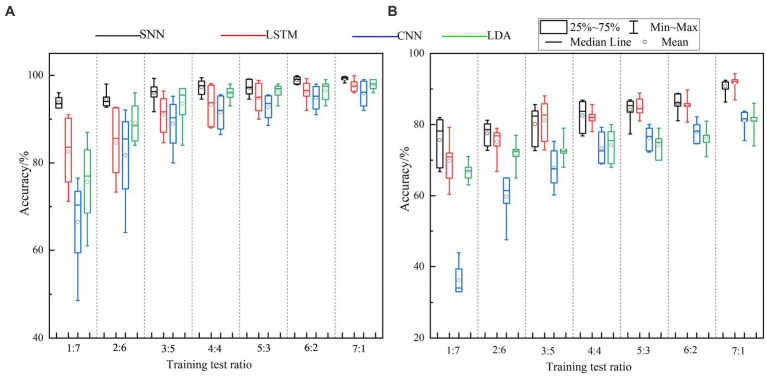
The recognition accuracies of **(A)** HD-sEMG and **(B)** LD-sEMG under different training test ratios.

This section conducts gesture recognition experiments on the HD-sEMG and LD-sEMG dataset under different training test ratios. The training test ratio is defined as the ratio of the number of gesture repetitions used to form the training set and the test set. In theory, the fewer gesture repetitions used to compose the training set, the smaller the user training burden. Concretely, the featured, real-valued, and spike samples are, respectively, divided into training and test sets according to the follow method: sequentially select *N* (1 ≤ *N* < 8) repetitions from eight repetitions to form the training set, and the remaining makes up the test set. The real-valued samples are input to LSTM and CNN, spike samples are input to SNN, and the featured samples are input to LDA. Figures 6A,B show the gesture recognition accuracies obtained from the HD-sEMG and LD-sEMG dataset, respectively. The results of One-Way Anova Analysis are reported in Table 4. According to the results, the following conclusions can be drawn:

**Table 4:** The one-way Anova analysis results of SNN with the other three networks under three kinds of experiments.

Gesture set	Multiple comparisons		Different training test ratio	Electrode-shift	User-independent
			Sig. (*p*) for accuracy		
HD-sEMG	SNN	LSTM	<0.0001***	0.865	0.286
		CNN	<0.0001***	0.474	0.126
		LDA	<0.0001***	0.014*	<0.001**
LD-sEMG	SNN	LSTM	0.288	0.442	0.997
		CNN	<0.001**	0.981	0.367
		LDA	<0.001**	0.059	0.312

***p < 0.0001, **p < 0.001, *p < 0.05.

First, SNN can effectively improve the gesture recognition accuracy. For the HD-sEMG dataset, compared with LSTM, CNN, and LDA, SNN significantly improves the recognition accuracies (*p* < 0.0001***). When the training test ratio varies from 1:7 to 7:1, the average recognition accuracy of SNN is 5.24, 9.23, and 4.73% higher than that of LSTM, CNN, and LDA, respectively. For the LD-sEMG dataset, compared with CNN and LDA, SNN significantly improves the recognition accuracy (*p* < 0.0001***), but there is no significant improvement compared to LSTM (*p* = 0.288). The average recognition accuracy of SNN is 0.99, 14.91, and 8.52% higher than that of LSTM, CNN, and LDA, respectively.

Second, SNN can effectively reduce the number of repetitions in the training set. When there is only one gesture repetition in the training set, SNN achieves the average recognition accuracies of 93.81 ± 1.87% for the HD-sEMG dataset and 75.71 ± 6.71% for the LD-sEMG dataset. However, for the HD-sEMG dataset, LSTM, CNN, and LDA only achieve the average recognition accuracies of 82.54 ± 7.87%, 66.45 ± 12.27%, and 75.62 ± 9.65% respectively, and for the LD-sEMG dataset, only achieve 69.73 ± 6.45%, 36.20 ± 4.44%, and 66.83 ± 2.78%, respectively. When there are more than three gesture repetitions in the training set, the average recognition accuracies of SNN are higher than 95 and 80% for two datasets respectively, however, CNN and LDA require at least seven gesture repetitions to achieve a similar performance.

### 3.5. Gesture recognition results of electrode-shift experiment and user-independent experiments

The electrode-shift experiment and user-independent experiment are carried out on all five trials of the HD-sEMG and LD-sEMG dataset. The electrode-shift experiment is carried out in a user-dependent mode. For each participant, the leave-one-out method is adopted to select one trial separately as the test set and the remaining trials as the training set. In the user-independent experiment, the leave-one-out method is used to select samples of each participant separately as the test set, and those of the remaining participants are used as the training set. Tables 4, 5 give the results of One-Way Anova Analysis and the average recognition accuracies for two experiments under all participants, respectively.

**Table 5:** The recognition accuracies (%) in electrode-shift and user-independent experiments.

Network	Electrode-shift		User-independent	
	HD-sEMG	LD-sEMG	HD-sEMG	LD-sEMG
SNN	72.59 ± 23.04	48.66 ± 16.29	66.61 ± 7.63	38.84 ± 7.03
LSTM	71.65 ± 23.95	51.73 ± 19.28	62.8 ± 6.62	37.73 ± 8.86
CNN	68.61 ± 24.09	48.51 ± 16.79	59.66 ± 6.75	35.77 ± 8.54
LDA	58.83 ± 23.19	41.4 ± 13.11	47.66 ± 9.56	35.25 ±6 .89

An overall observation of Tables 4, 5 shows that, in both electrode-shift experiment and user-independent experiment, the classification performance is only significantly improved compared with LDA in HD-sEMG datasets(*p* = 0.014*, *p* < 0.001**). Specifically, SNN performs better in HD-sEMG datasets. In the electrode-shift experiment of the HD-sEMG datasets, the average recognition accuracy of SNN is 72.59 ±23.04% , which is 0.94, 3.98, and 13.76% higher than those of LSTM, CNN, and LDA, respectively. However, for the LD-sEMG dataset, the average recognition accuracy of SNN is 48.66 ± 16.29% , which is 0.15 and 7.24% higher than those of CNN and LDA, but 3% lower than that of LSTM. In the user-independent experiment of HD-sEMG datasets, the average recognition accuracy of SNN is 66.61 ± 7.63%, which is 3.81, 6.95, and 18.95% higher than those of LSTM, CNN, and LDA, respectively. However, for the LD-sEMG dataset, the gap between the recognition accuracy of SNN and the other networks is reduced.

### 3.6. Performance comparison of different encoding methods and LIF neurons

As described above, adaptive threshold encoding is adopted to reduce the distribution differences of the EMG signals, and LIF neurons considering the voltage and current decay (LIF-V-I) are adopted to improve the feature extraction ability of SNN. In order to verify their superiority, comparative experiments are carried out on the HD-sEMG dataset. For the adaptive threshold encoding, fixed threshold encoding is used for comparison. When θ is in a stable range of (−0.2 ~ 0.6), the range of adaptive threshold $V_{thr1}$ is (0.08–0.2). Based on the range, the fixed thresholds are obtained in intervals of 0.02. By comparing the classification accuracy under different thresholds, the optimal fixed threshold $V_{thr1}$ is selected as 0.18. Adaptive threshold parameter θ is 0.6. As for the LIF-V-I, the LIF neuron (LIF-V) determined by Eqns. (5) and (6) is adopted for comparison. The $V_{thr2}\;$ of LIF-V is determined as 0.5 and the $V_{thr2}\;$ of LIF-V-I is 10. The electrode-shift and user-independent experiments are conducted with the following four schemes: adaptive threshold + LIF-V-I, fixed threshold + LIF-V-I, adaptive threshold + LIF-V, and fixed threshold + LIF-V. The division of the training and test sets is based on the leave-one-out method. From the experimental results shown in Table 6, it can be found that the adaptive threshold encoding and LIF-V-I neuron have relatively superior performance. Under electrode-shift and user-independent experiments, compared to the fixed threshold encoding, the adaptive threshold encoding achieves (3.39–6.75%) improvement when LIF-V-I and LIF-V are used. Compared to LIF-V neurons, LIF-V-I achieves (1.31–2.31%) improvement when the adaptive threshold encoding and the fixed threshold encoding are used. Compared to fixed threshold + LIF-V, adaptive threshold encoding + LIF-V-I achieves 5.24 and 8.06% improvement.

**Table 6:** The recognition accuracies (%) under four schemes.

Methods	Electrode-shift	User-independent
Adaptive threshold + LIF-V-I	72.59 ± 23.04	66.61 ± 7.63
Fixed threshold + LIF-V-I	69.20 ± 24.63	59.86 ± 8.56
Adaptive threshold**+** LIF-V	70.80 ± 26.32	64.30 ± 6.89
Fixed threshold**+**LIF -V	67.35 ± 29.55	58.55 ± 8.55

## 4. Discussion

Low robustness, heavy training burden, and high power consumption are important factors that hinder the application of myoelectric control technology. Based on the experimental results presented in the above sections, the feasibility and limitations of applying the SNN-based myoelectric pattern recognition scheme proposed in this study to actual myoelectric control systems can be discussed as follows.

### 4.1. The validity of the proposed SNN scheme in reducing power consumption

It is well known that floating-point matrix multiplication of DNN is the fundamental reason for high power consumption. Liu et al. demonstrated the first real-time embedded gesture recognition system composed of three feedforward ANN layers, which can recognize 10 gestures with a processing power consumption of 69mw (Liu et al., 2016). Benatti et al. combined parallel ultra-low power platform (PULP) using binary hyper-vectors with a brain-inspired algorithm, achieving an average accuracy of 85% for an 11-gesture recognition task and an average power of 10.4 mw for a classification (Benatti et al., 2019). However, the hyper-vectors are often dense and have huge dimensions, making a further reduction in power consumption difficult. Since the event-driven mechanism of SNN enables floating-point numbers to be replaced by sparse binary vectors without dimension expansion, SNN has greater advantages in reducing power consumption. Donati et al. implemented a SNN on a multicore neuromorphic chip and obtained 74% recognition accuracy for a 3-gesture recognition task (Donati et al., 2019). The average power for a classification was 0.05 mw, which is 1/208 of the power consumption of PULP and 1/1380 of the feedforward ANN.

In this study, the inference power of SNN is positively correlated with SRR . As shown in Figures 5A,B, when $\bar{SRR_{1}}$ and $\bar{SRR_{2}}$ ranges are within a stable range, satisfactory recognition accuracies can be obtained. Therefore, we can use minimum SRR of the stable range to further reduce the power consumption. According to Table 3, the power consumption of LSTM and CNN are about 1–2 orders of magnitude higher than that of SNN, which is consistent with the power consumption measured by the above literature. In addition, SNN also has lower latency compared with LSTM and CNN. Therefore, the lower power consumption and lower latency of SNN makes it more suitable than DNN for real-time gesture recognition in myoelectric control systems.

### 4.2. The validity of the proposed SNN scheme in reducing user training burden

Because of the individual differences of physiological signals among users, enough training data is usually collected to train a specific classifier for each user. For instance, Phinyomark et al. achieved 92.11% accuracy for a 6-gesture recognition task using an LDA and nine gesture repetitions for training and a repetition for testing (Phinyomark et al., 2012). Li et al. trained a generalized network on a dataset containing 30 gestures and used migration learning to reduce training burden (Chen et al., 2021). The target network achieved 90% recognition accuracy in the 30-gesture, 10-gesture, and 8-gesture recognition tasks, respectively, using over two gesture repetitions for training. For a 5-channel 8-gesture task, Cheng et al. achieved 97.4% accuracy using a pre-trained SNN trained with 45 gesture repetitions (Cheng et al., 2021). For an 8-channel 3-gesture task, Ma et al. implemented a SRNN (Ma et al., 2020). When 12 gesture repetitions were adopted for training and three gesture repetitions for testing, the classification accuracy exceeded 85%. For 10-channels 3-gesture recognition tasks, Tian et al. used spiking-CNN to achieve 85.7% gesture recognition accuracy using eight repetitions for training and two repetitions for testing (Tian et al., 2023).

Some studies have verified the superiority of SNN for reducing training burden in other applications. Ma et al. (2018) used SNN to classify the MNIST dataset, which contains 10 kinds of handwritten digital pictures, consisting of 60,000 training samples and 10,000 test samples. When all training samples were used for training, the recognition accuracy of SNN and CNN were 90.44 and 98.19%, respectively. However, when only 1,000 training samples were used, the accuracy of SNN was still as high as 80.15%, whereas that of CNN was only 10.28%.

In this study, we examine the performance of SNN on HD-sEMG and LD-sEMG datasets under different user training burdens. To the best of our knowledge, this is the first exploration on training burden of SNN-based myoelectric pattern recognition. The experimental results in Figure 6 and Table 4 show that SNN can achieve high-accuracy myoelectric pattern recognition under lower training burden. SNN achieved higher recognition accuracy using only three gesture repetitions for training, compared to six repetitions required by LSTM, and seven repetitions required by CNN and LDA. Even when only one gesture repetition is used for training, the SNN achieves average recognition accuracies of 93.81 ± 1.87% and 75.71 ± 6.71%for the HD-sEMG and the LD-sEMG dataset respectively, which is about an 11.27 and 5.98% improvement over LSTM, about 27.36 and 39.51% improvement over CNN, and about 18.19 and 8.88% improvement over LDA. In addition, for the HD-sEMG dataset, the recognition accuracy of SNN can partly exceed that of existing works both in terms of training burden and recognition accuracy. For 8-channel EMG signals, the recognition accuracy is also comparable to other works based on SNN. Therefore, this study proves that only a small amount of data needs to be collected to train a satisfactory SNN classifier, which provides the possibility for the realization of myoelectric control systems with low user training burden.

### 4.3. The performance of the proposed SNN scheme in improving robustness

There are two main factors that have a great impact on the robustness of myoelectric control systems, which are electrode shifts caused by electrode replacement or human movement and individual differences in physiological signals. First, the use of HD electrodes combined with the spatiotemporal feature extraction ability of DNN is often used to solve the above problems. Meng et al. constructed 1D-CNN, 2D-CNN, and CNN-LSTM to carry out the classification task among users on the 256-channel sEMG datasets of 10 gestures (Meng et al., 2022). Compared with SVM, the recognition accuracies of three networks increased significantly. Second, specific training strategies are usually designed to ensure the robustness of myoelectric pattern recognition. For instance, Vidovic et al. proposed a pre-trained hybrid LDA for the recognition of eight gestures (Vidovic et al., 2016). The parameters of the pre-trained LDA model were calibrated using EMG data with shifts, and the classification accuracy reached more than 92%. Côté-Allard et al. (Cote-Allard et al., 2020) introduced a new multi-domain learning algorithm, named ADANN, for implementing an inter-subject classification task. Designing training strategies often requires collecting extra training data for training or fine-tuning, which will increase heavy training burden. Third, the advanced algorithms are designed to solve electrode shifts and individual differences. For electrode shifts, Hu et al. proposed an adaptive electrode calibration method using a fast-independent component analysis algorithm to extract the muscle core activation region for gesture recognition (Hu et al., 2021). For an inter-subject task, Xue et al. proposed a framework based on typical correlation analysis and optimal transmission (OT), called CCA-OT (Xue et al., 2021).

This paper is the first work to explore the performance of SNN in solving the problems of electrode shifts and individual differences. Aiming to ensure the robustness of the SNN, we employ the adaptive threshold encoding to weaken the distribution differences of EMG data and use LIF-V-I neurons to improve feature extraction ability. The results of Table 6 indicate that these two strategies are effective. According to Tables 4, 5, overall, SNN has advantages in improving recognition accuracies in both the electrode-shift experiment and user-independent experiment. Since SNN can achieve even exceed the robustness of existing DNN with low power consumption and significantly outperform the robustness of LDA, we conclude that the proposed SNN-based scheme has more advantages in the application of myoelectric control systems.

### 4.4. Limitations and future works

We would like to point out the limitations of the current research. First, this study only selects a single time-contrast encoding method and the threshold-emitting neuron model to construct the SNN. Further attempts should be made to try more encoding algorithms and neuron models; second, the EMG samples in this study are obtained only from the stabilization phase of gesture execution, and the transitions between rest and gesture execution may be a factor affecting the robustness of myoelectric pattern recognition, which should be explored in the future; third, the proposed SNN-based scheme should be further verified on various publicly datasets; fourth, although the proposed SNN has certain advantages over simple CNN, LSTM, and LDA in terms of electrode-shift and user-independent tasks, the gesture recognition accuracy obtained by SNN is far from that obtained by related studies. In the work of Hu et al. (2021) using the same database, the gesture recognition accuracy under electrode shifts was 92.17%, whereas the accuracy in this study is only about 72%. With reference to the recent research progress in electrode shifts and individual differences, advanced training strategies and signal processing algorithms should be introduced into SNN in the future; finally, the proposed SNN is carried out offline on a computer, and the measurement for network power consumption and inference delay is an algorithmic estimation, ignoring the potential problems of hardware implementation. In the future, it should be implemented on neuromorphic circuits.

## 5. Conclusion

This study is the first to explore the feasibility of applying SNN to myoelectric control systems from the aspects of training burden, robustness, and power consumption. Its main work and contributions are as follows: (1) Taking SNN as the basic network architecture, the adaptive threshold temporal contrast encoding and the LIF neuron that combines voltage–current effects are applied to improve the performance of SNN. Meanwhile, the adaptive threshold encoding parameter and LIF neuron release threshold are determined by experiments to balance recognition accuracy and power consumption as much as possible and (2) By conducting myoelectric pattern recognition experiments on the HD-sEMG and LD-sEMG in the cases of different training test ratios, electrode shift, and user independence, the advantages of SNN in reducing power consumption, alleviating training burden, and improving the robustness have been verified. The research results of this study are of great significance for the implementation of user-friendly low-power myoelectric control systems.

## Data availability statement

The raw data supporting the conclusions of this article will be made available by the authors, without undue reservation.

## Ethics statement

The studies involving human participants were reviewed and approved by the Ethics Review Committee of First Affiliated Hospital of Anhui Medical University (No. PJ 2014-08-04). The patients/participants provided their written informed consent to participate in this study.

## Author contributions

AS designed the research scheme, did the data processing, analysis, and gesture recognition experiments, and wrote the manuscript. XiC directed the research and substantially revised the manuscript. MX participated in the design of the research scheme. XZ and XuC participated in the interpretation of the research results and manuscript revision. All authors contributed to the article and approved the submitted version.

## Funding

This work was supported by the National Natural Science Foundation of China under grant 82272113 and 61871360.

## Conflict of interest

The authors declare that the research was conducted in the absence of any commercial or financial relationships that could be construed as a potential conflict of interest.

## Publisher’s note

All claims expressed in this article are solely those of the authors and do not necessarily represent those of their affiliated organizations, or those of the publisher, the editors and the reviewers. Any product that may be evaluated in this article, or claim that may be made by its manufacturer, is not guaranteed or endorsed by the publisher.
